# A Novel Miniaturized Biosensor for Monitoring Atlantic Salmon Swimming Activity and Respiratory Frequency

**DOI:** 10.3390/ani11082403

**Published:** 2021-08-14

**Authors:** Jelena Kolarevic, Josep Calduch-Giner, Åsa M. Espmark, Tor Evensen, Javier Sosa, Jaume Pérez-Sánchez

**Affiliations:** 1 Nofima, Muninbakken 9-13, Breivika, 9019 Tromsø, Norway; jelena.kolarevic@uit.no (J.K.); Asa.Espmark@nofima.no (Å.M.E.); Tor.Evensen@Nofima.no (T.E.); 2 Nutrigenomics and Fish Growth Endocrinology Group, Institute of Aquaculture Torre de la Sal, Consejo Superior de Investigaciones Científicas (CSIC), 12595 Castellón, Spain; j.calduch@csic.es; 3 Institute for Applied Microelectronics (IUMA), University of Las Palmas de Gran Canaria, 35015 Las Palmas, Spain; jsosa@iuma.ulpgc.es

**Keywords:** Atlantic salmon, welfare monitoring, swimming activity, respiration frequency, biosensors

## Abstract

**Simple Summary:**

Good fish welfare is one of the prerequisites for sustainable aquaculture. Knowing how fish respond to the production conditions would allow us to better understand their biology and to further optimize production. The new miniaturized biosensor AEFishBIT was successfully used to monitor individual physical activity and respiratory frequency of two Mediterranean farmed fish species (gilthead sea bream and European sea bass). In this study, we aimed to test the use of AEFishBIT to monitor the performance of Atlantic salmon under experimental conditions. An adapted tagging procedure for salmon was developed and used to record salmon responses to handling and changing light conditions. AEFishBIT data showed a stabilization of swimming activity 8 h after handling and tagging with changes in activity or activity and respiratory quotient after changes in light intensity regimes. The results of this study supported the use of AEFishBIT to generate new behavior insights in Atlantic salmon culture.

**Abstract:**

The advanced development of sensor technologies has led to the emergence of fish biosensors that are currently used for research and commercial purposes. AEFishBIT is a miniaturized biosensor attached to fish operculum that measures physical activity and respiration frequencies. In this study, we determined the effect of the tagging method and evaluated the use of this biosensor to monitor post-smolt Atlantic salmon in a tank-based system. The use of piercing fish tag had a negative impact on the gills and operculum, unlike the identical protocols used in gilthead sea bream and European sea bass. In contrast, a surgical thread did not show any apparent tissue damage. Two data recording schedules were considered to monitor immediate early reaction to fish handling and light regime changes (records every 15 min over 2 days) or adaptation to new light conditions (records every 30 min over 4 days). Data showed stabilization of physical activity 8 h post-tagging, with different steady states for the activity/respiratory ratio after changes in light intensity that reflected a different time course adaptation to new light conditions. High correlations were observed between AEFishBIT and video recording data. These findings supported the use of AEFishBIT as a promising tool for smart sensing of Atlantic salmon.

## 1. Introduction

Aquaculture is facing a technological transformation from being a traditional labor-intensive to a mechanized and automated food production industry [1,2]. Biosensor technology can transform the aquaculture industry by collecting data that will be informative on fish growth and welfare status, environmental parameters, operation, and resource allocation [3,4,5]. This approach is the basis of precision aquaculture, which will facilitate increased production in accordance with more ethical and sustainable fish farming in a friendly environment [6]. This also involves measurements of whole-organism traits related to behavior, health, welfare, and metabolic status.

A range of welfare indicators, both environmental and animal-based, are available to assess welfare under experimental and commercial conditions [7]. Some of them are more suitable for use during on-farm welfare assessment and are termed operational welfare indicators (OWI) [8], while others, named laboratory-based welfare indicators (LABWI), require the use of laboratory or other remote analytical facilities [7]. In any case, most of the animal-based welfare indicators currently in use require fish to be removed from their environment and, in some cases, sacrificed to obtain the necessary information on current welfare status. The use of biosensor technology can allow fish status to be monitored for a longer time and can generate knowledge about the causes of changes in welfare status, which is the important step towards optimizing culture conditions.

Fish behavior is a reliable group-based welfare indicator that can serve as an early warning sign of stress or other factors that threaten welfare [9]. For both research and industry purposes, video recordings provide a consistent overview of fish behavior. Many farms use submerged video cameras in cages to monitor feeding behavior and position of fish, and to adjust feeding accordingly. Quantification of behavioral traits using video recordings for research questions provides detailed information on different behavioral patterns, reactions to stimuli, and deviations from what is considered normal behavior [10,11]. However, the analysis of behavioral data in aquaculture is time consuming and can be subjective. Additionally, monitoring individual behavior would require fish to be visibly ID-tagged and clearly visible on video recordings. Turning quantitative behavioral analysis to reliable OWI will require technological advances such as the use of machine vision solutions or biotelemetry and bio-loggers [7].

Acceleration data-loggers, alone or in combination with pressure, temperature, and heart rate biosensors, have been used to track movement and estimate activity-specific energy expenditure in a number of fish species, including juvenile hammerhead sharks (*Sphyrna mokarran***)**, sockeye salmon (*Oncorhynchus nerka*), European sea bass (*Dicentrarchus labrax*), Atlantic cod (*Gadus morhua*), Atlantic salmon (*Salmo salar*), and red-spotted groupers (*Epinephelus akaara*) [12,13,14,15,16,17]. These sensors generally operate as acoustic transmitter tags that contain a tri-axial accelerometer, which records the forces of gravity and acceleration in the *X*-, *Y*-, and *Z*-directions. However, interference between the transmitted signals limits the use of a large number of acceleration tags in a single rearing unit or in two neighboring units. Otherwise, in the aquatic environment, the use of low radiofrequency transmission is limited to 1–2 m of the maximum communication range without repeaters.

With the dual objective of combining measurements of metabolism and behavior of farmed fish in a laboratory-based environment, a stand-alone ultra-low power device was designed and produced within the AQUAEXCEL^2020^ EU project using commercial components and already available technology. The resulting device (AEFishBIT) was patented (PCT/ES2019/070205) as a stand-alone, small, and lightweight (1 g) motion embedded-microsystem to be used as a tri-axial accelerometer that is attached to the operculum to monitor physical activity by mapping of the accelerations in *X*- and *Y*-axes, while the operculum beats (*Z*-axis) serve as a measure of respiratory frequency [18,19]. This device is aimed to be used at laboratory scale for rapid and precise individual fish phenotyping of behavioral traits of interest, as part of the new generation of technology-based procedures for the improvement of welfare and selective breeding in aquaculture. Data are processed by on-board algorithms, and the device is recovered at the end of each recording period to recharge the battery recharging, program schedules, and download data. The accuracy of on-board algorithms was evaluated with exercised gilthead sea bream (*Sparus aurata*) and European sea bass in a swim tunnel respirometer, the outputs of the accelerometer being highly correlated with swimming speed and oxygen consumption [18]. As part of the validation procedure, changes in the amplitude and frequency of operculum and body tail movements highlighted the adaptive anatomical and metabolic features of European sea bass as a fast-swimming predator [19]. In addition, the use of AEFishBIT in free-swimming gilthead sea bream clearly stated that the simultaneous monitoring of locomotor activity and respiratory frequency is a reliable tool for individual monitoring of whole-fish traits in fish challenged with a wide range of biotic and abiotic factors (e.g., genetic background, age, space availability, feeding time and frequency, oxygen availability, thermal stress, disease progression), and patterns arising from AEFishBIT measurements are associated with better performance or differences in stress response and disease resilience [20,21]. The impact of device operculum attachment was also evaluated in this fish species, and no signs of growth impairment or damage to the operculum or gills were found. Thus, AEFishBIT opens up new research opportunities for the individual phenotyping of economically important fish, serving to establish stricter welfare criteria and to improve selective breeding in a scenario of global change, where reduced oxygen availability, alternative feed formulations, and disease weakness are becoming major aquaculture stressors around the world.

The aim of the present study was to validate the use of AEFishBIT in another highly cultured species of economic importance to the world aquaculture industry, the Atlantic salmon. To do that, we first evaluated the impact of operculum tagging on post-smolt salmon by tracking different morphological OWI. In addition, AEFishBIT was used to assess physiological status and swimming activity after handling and changes in lighting, comparing biosensor and video recording data for functional validation purposes.

## 2. Materials and Methods

### 2.1. Experimental Fish

Atlantic salmon post-smolts were produced at the Nofima research station at Sunndalsøra, Norway from eggs (AquaGen strain). Prior to the tagging and data recording experiments, fish were placed for at least two months in 3.3 m^3^ octagonal tanks with full strength sea water (34 ppt). During that time, fish were reared in a continuous light regime, feed was offered in excess once per hour, oxygen levels were maintained >85% saturation, water temperature was 9.0 °C, and water flow was kept in the range of 110–120 L·min^−1^. The study adhered to the guidelines and protocols of European Union Directive 2010/63/EU and the National Guidelines for Animal Care and Welfare established by the Norwegian Ministry of Education and Research. The experiment was approved by the Norwegian Food Safety Authority under FOTS ID 22193. Key personnel of the fish trial hold a FELASA C certificate.

### 2.2. Assessment of AEFishBIT Tagging Impact

**Figure 1:**
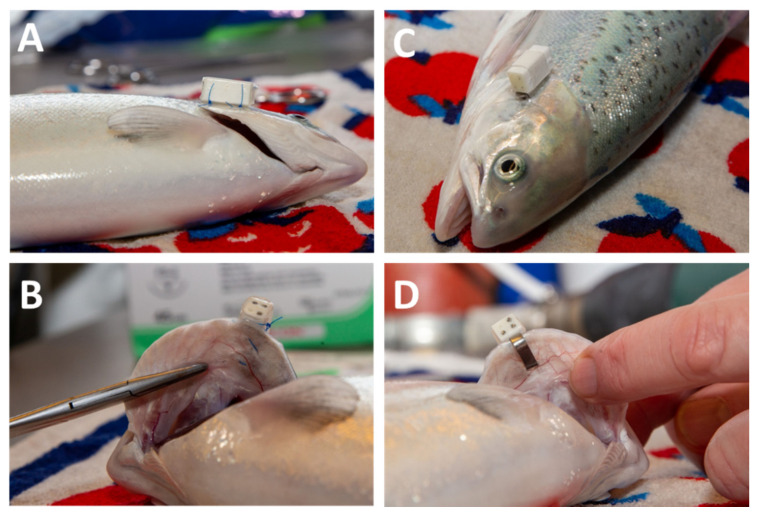
AEFishBIT biosensor attached to Atlantic salmon (*Salmo salar*) operculum using surgical thread (**A**,**B**) and Self-Piercing fish tag (**C**,**D**).

**Figure 2:**
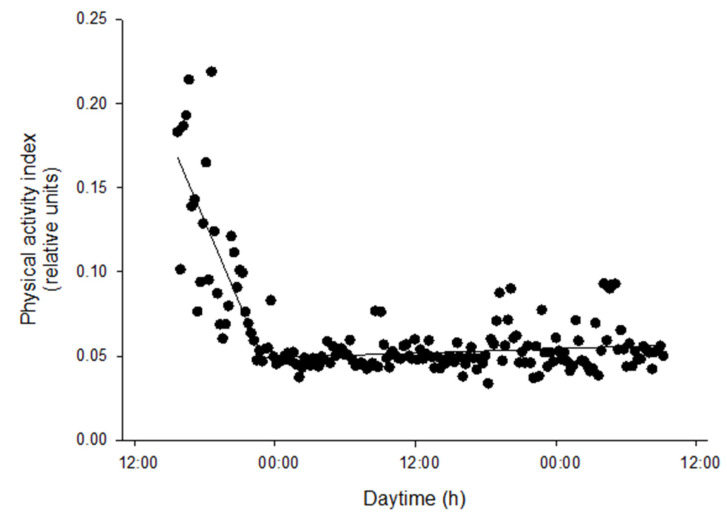
AEFishBIT records of physical activity of a representative individual after tagging and return to the tank. Measures (black circles) were taken along two consecutive days every 15 min. Lines represent piecewise linear fit of data with one break point.

A preliminary test (pilot study) with post-smolts was conducted to monitor the impact of two tagging methods for external attachment of AEFishBIT to the salmon operculum. Fish were kept in a 3.3 m^3^ tank and acclimatized to a density of 27 kg·m^−3^. A total of 40 fish (20 fish for tagging method) with an average body weight of 456 ± 98 g were tagged with dummy PA 2200 polyamide tags, with the same dimensions (15 × 6 × 6 mm) and weight (1.1 g in air) than the functional AEFishBIT. Post-smolts were tagged as follows: (1) the biosensor was held in place with a ring made of flexible heat shrink polyethylene tubing, which was sutured with 1 or 2 surgical threads in the operculum (Figure 1A,B); (2) the biosensor was pasted onto a Self-Piercing Fish Tag-Style 1005-3 (3/16″H × 7/16″L inside dimensions, National Band & Tag Company, Newport, CA, USA) and attached to the operculum (Figure 1C,D) as previously reported in gilthead sea bream and European sea bass [19,20]. In both cases, fish were individually placed in a bucket with rearing water and transferred to the work station for tag attachment. Fish were then placed in a bath with 110 mg·L^−1^ tricainemethane sulphonate (MS-222; FINQUEL^®^vet, ScanAqua AS, Årnes, Norway) until loss of balance and respiratory arrest were observed. Individuals were removed from the water, weighed, and placed on a wet towel during tagging. After tagging, fish were released into a recovery tank with clean water until equilibrium was recovered. After returning to the rearing tank, the behavior and appetite of the tagged fish were monitored daily for one week, as well as the survival and tag retention rates.

### 2.3. Light Intensity Challenge

At the beginning of the experiment (D0), 196 fish from one tank with fluorescent light (average light intensity of 9.43 μmol·m^−2^·s^−1^) were divided between three 3.3 m^3^ octagonal tanks (11.6 kg·m^−3^) with the same type of lighting. The water flow in the tanks was 66 L·min^−1^, and the average temperature during the study was 10.3 ± 0.1 °C. Sixteen fish were tagged per tank with functional AEFishBIT devices using the polyethylene tube sutured with surgical thread procedure. For recorded video footage, AEFishBIT-tagged fish were additionally tagged with yellow numbered Peterson’s discs (P-disc; Floy Tag & Mfg., Inc., Seattle, WA, USA) of appropriate size. Each P-disc consisted of two discs that were connected by a nickel pin or a thread. One disc was placed under the dorsal fin and connected subcutaneously with the nickel pin or thread to identical disc placed on the other lateral side of the fish. An additional 48 fish were tagged only with red numbered P-discs to assess the specific effects of AEFishBIT tagging on morphological OWI during the recording period.

Fish were allowed to recover from tagging and handling until the morning of D1 when the lighting conditions in the tanks were changed at 09:00 h (Table 1): one tank was switched to full spectrum LED light and “high” average light intensity (HI treatment; light intensity 18.90 μmol·m^−2^·s^−1^), a second tank to full spectrum LED light and “low” average light intensity (LI treatment; light intensity 0.28 μmol·m^−2^·s^−1^), while a third tank remained with the same fluorescent light and served as a control group (C treatment; light intensity 6.83 μmol·m^−2^·s^−1^). Light intensity measurements were previously performed in all tanks using the LI-COR LI-193 spherical underwater quantum sensor and LI-1500 light sensor logger (Li-Cor, Inc., Lincoln, NE, USA).

AEFishBIT devices were programmed for on-board calculation of respiratory frequency and physical activity. The program schedule for eight devices per tank was 2 min time measurement windows every 15 min during days D0 and D1. This program was designed to evaluate salmon behavior immediately after handling and tagging. The remaining eight devices per tank had 2 min time measurements windows every 30 min for 4 experimental days (from D0 to D3) for extended behavior evaluation after the change of lighting conditions on day D1. Video recordings were made with GoPro cameras between 08:55 h and 10:05 h on D1 and D3 (see Section 2.5) to determine the behavior immediately after the change in lighting conditions, as well as on the last day of experiment. During the entire experimental period, fish were fed once every hour at predetermined time points (every 8th minute in the hour) under a continuous light regime. At D3, fish were euthanized with an overdose of MS-222, AEFishBIT devices were retrieved, and processed data were downloaded.

### 2.4. AEFishBIT Data Acquisition

Sampling frequency of AEFishBIT was 50 Hz, and software processing of the raw accelerometer data estimated respiratory frequency and physical activity following the procedures described in [18,19]. Briefly, the *Z*-axis raw data consisted of a periodic oscillatory signal that followed the opening and closing of the operculum. Other movements superimposed on this *Z*-axis signal were angular accelerations related to fish trajectory or side head movement, although the dominant periodic component was the operculum movement. To estimate the respiratory frequency, we integrated the accelerometer *Z*-axis signal to obtain the velocity and bandpass filtered between 0.5 and 8 Hz to reduce noise, attenuate the influence of other movements, and highlight the periodic properties of the signal. Subsequently, the number of crosses through zero was divided by two to obtain the period of the signal. This value, averaged over several frames over 2 min to reduce bias, corresponded to the estimated respiratory frequency. To describe physical activity, we relied on the simple optimization principle that human movements try to perform maximally smooth movements by minimizing the first temporal derivative of acceleration, or “jerk” while constraining all higher derivatives to zero [22]. This fact has also been applied to measure the intensity of the movement of livestock [23], and herein the averaged *X*-axis and *Y*-axis accelerations were derived to obtain the jerk, and their averaged energy over 2 min was considered representative of physical activity. For each device, the clock time drift was pre-estimated for post-processing synchronization. This time drift was established as constant for a given device in a temperature range of 4–30 °C.

### 2.5. Video Recording and Analyses

In total, 1 h surface video recordings were taken at the same time (9:00–10:00 h) during the morning of D1 and D3. The recording time was selected to minimize personnel traffic where the recordings were taken, and to film the response of fish to the change in lighting. From the video recordings, the same 5 min interval of all tanks (minutes 5–10) was analyzed. The video recording analyzed untagged fish, fish tagged with P-disc, and fish tagged with P-disc and AEFishBIT. To determine Atlantic salmon reaction to the light change, we determined the proportion of time that fish spent swimming before and after the light change during D1.

### 2.6. Monitoring of Morphological Welfare Indicators

Fish weight and length were measured at D0 and D3. In parallel, several OWI were evaluated to assess the impact of AEFishBIT tagging compared to other external tags. Briefly, 12 morphological indicators were examined and scored from 0 to 3, where score 0 marked no change and 3 marked severe change for each OWI [7]. Special attention was paid to visual examination of gills, operculum, wounds, inflammation, and/or irritation at the location of the tag. A visual inspection of the intestinal content was also carried out at the end of the experiment to check if fish had regained feeding.

### 2.7. Statistical Analysis

Processed AEFishBIT data were analyzed by one-way ANOVA followed by Holm-Sidak *post-hoc* test when more than two groups were compared, and Pearson correlation coefficients by means of the Sigmaplot suite (Systat Software Inc., San Jose, CA, USA). Behavioral data were analyzed using one-way ANOVA.

## 3. Results

### 3.1. Impact of AEFishBIT Tagging Procedure

All tagged individuals in the pilot study recovered successfully, and there was no mortality throughout the experimental period. None of the fish with dummies showed abnormal behavior, and all displayed a correct position in the tank (Figure S1). One week post-tagging, the retention rate of dummy tags attached with the Self-Piercing tag was 100%, while the retention rate of the sutured tags was 85%. Three of the 20 tags were recovered from the feed collectors on days 5, 6, and 7. Regarding fish with a Self-Piercing tag, 19 individuals showed signs of gill lamellae damage that included loss of tissue at the contact points between piercing tag and gill, as well as discoloration. Irritation and damage to the opercular tissues was observed at the point where the Self-Piercing tags were attached. The most severe damage was found in individuals smaller than 350 g. Fish with sutured AEFishBIT tags had no visible gill damage, and only 10% individuals had minimal signs of inflammation at the tag attachment point one week post-tagging.

### 3.2. Atlantic Salmon Response to Handling

Immediately after fish handling, AEFishBIT recordings of the lighting challenge highlighted high levels of physical activity, with values of 0.23 relative units, which decreased to stabilization (0.04–0.10 relative units) approximately 8 h post-tagging (Figure 2). This stabilization was not dependent of the light regime changes, which occurred later at 09:00 h of D1. 

### 3.3. Transient Response to Change in Light Intensity in Rearing Tanks

**Figure 3:**
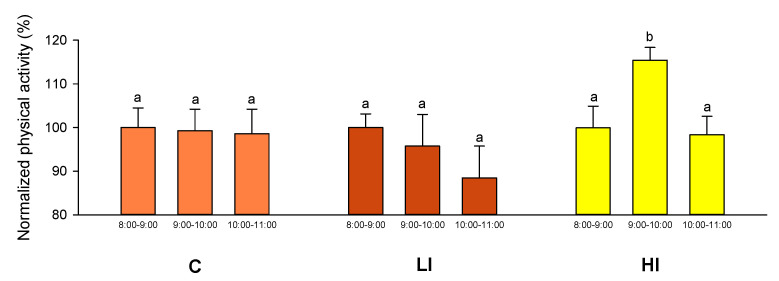
AEFishBIT records (D1) of reaction to change in light regime. Light was changed at 9:00 h (D1) in the LI (red bars) and HI (yellow bars) groups, whereas it remained unchanged for the C (orange bars) group. For each experimental group, physical activity is referred as a percentage of measured values in the 8:00–9:00 h period (before light change). Each value is the mean ± SEM of 6–7 individuals. Different letters indicate significant differences (*p* < 0.05, one-way ANOVA followed by Holm–Sidak test).

AEFishBIT monitoring of physical activity on D1 highlighted the reaction of experimental groups to the change of light regime (Figure 3). For the C group, there were no differences among individuals between their mean physical activity indexes in the 8:00–9:00 h and 9:00–10:00 h periods. This same pattern was observed for the LI group, where the light regime change occurred at 09:00 h. On the contrary, fish of the HI group showed a significant increase in the 09:00–10:00 h period compared to the previous hour in concurrence with the change in light regime, returning physical activity to values prior to the change in light regime in the following hour (10:00–11:00 h period).

**Figure 4:**
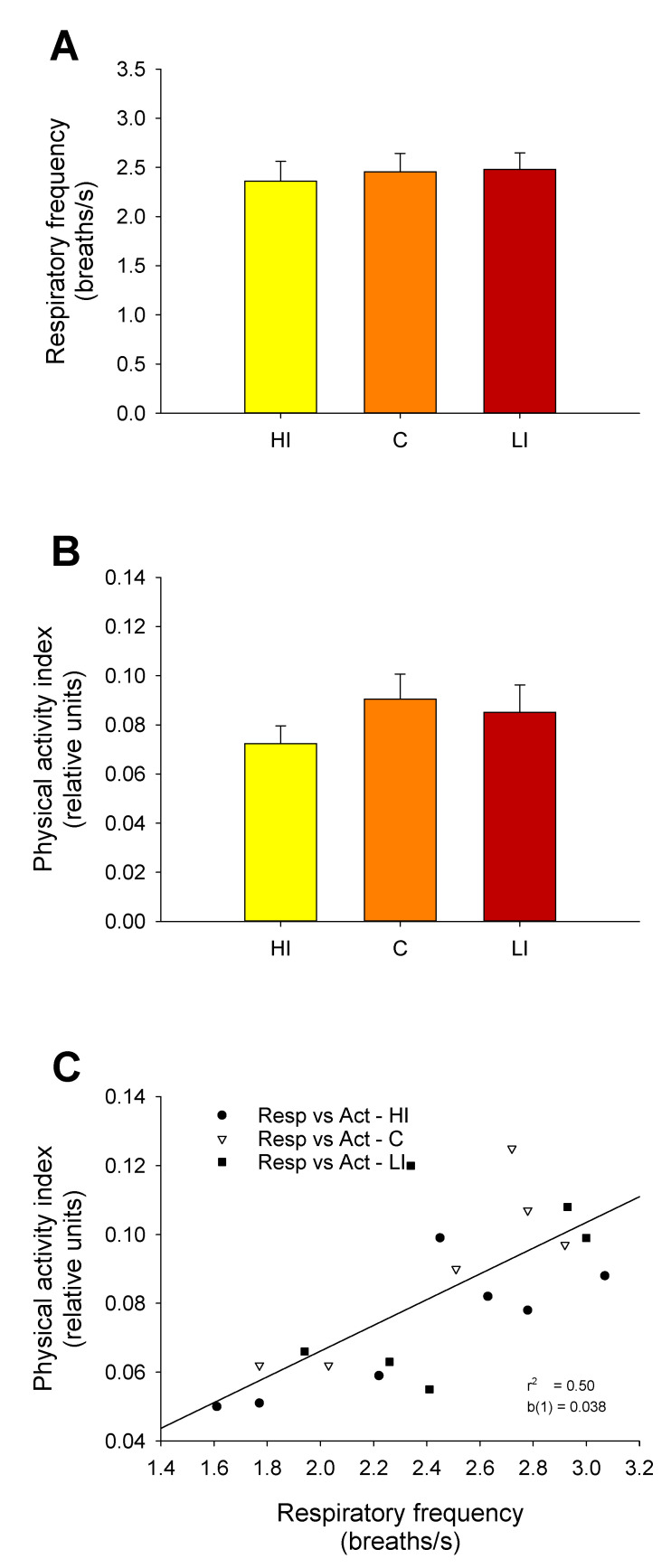
Daily averaged AEFishBIT records of respiratory frequency (**A**) and physical activity (**B**) after three days of exposure (D1–D3) to different light intensities: HI (yellow bars), C (orange bars), and LI (red bars). Each value is the mean ± SEM of 6–7 individuals. (**C**) Linear correlation between respiratory frequency and physical activity of fish individuals exposed to HI (circles), C (triangles), or LI (squares) lighting.

After changes in light regimes, no differences were found among experimental groups in mean respiratory frequency or physical activity during D1 to D3 (Figure 4A,B). For physical activity, a trend to decreased levels was observed in the HI group, although it was not statistically significant. When the data of all AEFishBIT-tagged individuals were grouped together, a positive linear correlation was evidenced between the respiratory frequency and the physical activity index (Figure 4C).

**Figure 5:**
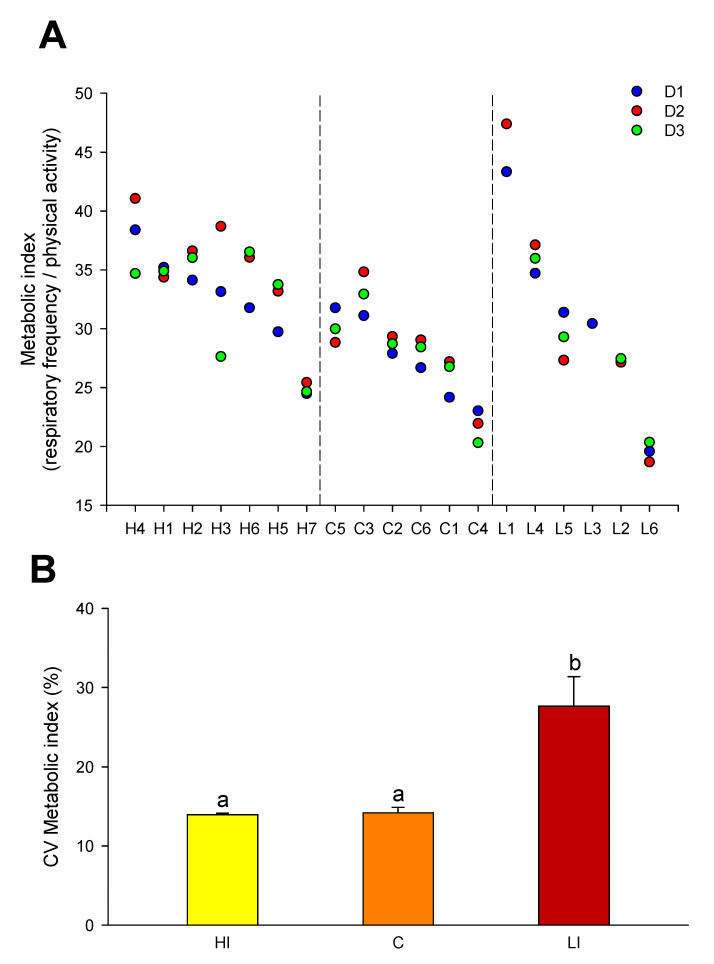
(**A**) Daily metabolic index (respiratory frequency/physical activity) in each individual during three days of exposure to different light intensities (D1–D3): HI, C, and LI. For each individual, the metabolic indexes of D1 (blue circles), D2 (red circles), and D3 (green circles) are represented. (**B**) Coefficient of variation (CV) of the metabolic index of fish exposed to different light intensities: HI (yellow bars), C (orange bars), and LI (red bars). Each value is the mean ± SEM of the daily coefficient of variation of 6–7 individuals for three days. Different letters indicate significant differences (*p* < 0.01, one-way ANOVA).

The quotient between the values of respiratory frequency and physical activity was used as a metabolic index, with large differences in the dispersion of data among groups (Figure 5A). This was evidenced by changes in the coefficient of variation that was similar in fish of HI and C groups, but significantly higher in fish of LI group (Figure 5B). 

### 3.4. Correlation between AEFishBIT and Video Recording Data

**Figure 6:**
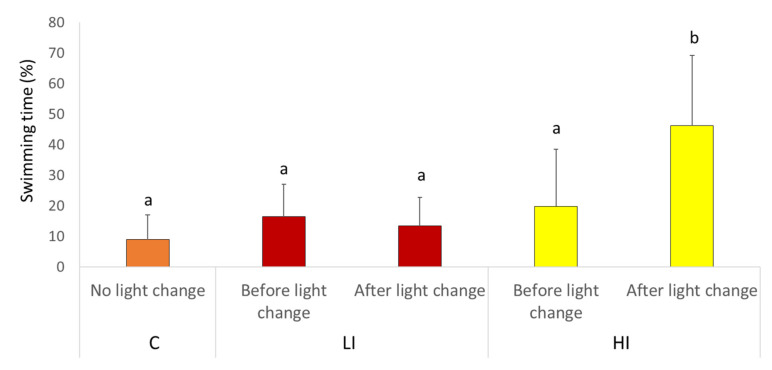
Swimming time (%) before and after switch in light (D1). Data are expressed as means ± SD and tested with one-way ANOVA (*p* < 0.001). Small letters show significant differences between the groups. *N* = 15 for C, LI, and HI groups.

Video recording analysis did not detect differences among the tagging groups (P-disc, P-disc and AEFishBIT, and untagged fish) for the C, LI, and HI lighting regimes. Therefore, the different tagging groups were merged for each light regime and behavior monitoring. The percentage of swimming time of individuals immediately after the light regime change was higher when switching light from control to full spectra LED with LI in comparison to C group (Figure 6). This pattern was in agreement with that determined by the AEFishBIT records (HI group in Figure 3).

Video monitoring also evaluated the response of fish to feeding on D1. There were no differences between the light regime groups in terms of feed interest considering the response latency time, although the percentage of fish that did not show interest in feed was higher in the HI group (50%) than C and LI groups (29% and 31%, respectively) (Figure S2).

### 3.5. Atlantic Salmon Performance and External Morphological Welfare Indicators

In the lighting challenge, the initial weight of salmon tagged with AEFishBIT was 786 ± 108 g, while fish tagged with only P-discs weighed on average 736 ± 162 g. The final mean weights for fish with AEFishBIT and with P-disc were 780 ± 112 g and 722 ± 159 g, respectively. Only 12.5% of fish with P-discs gained weight, while 33.3% of fish additionally tagged with AEFishBIT had the same weight or increased weight at the end of the recording period.

A visual inspection of the gut content of all fish groups exposed to HI and LI treatments was carried out to determine if fish had regained feeding after the experiment. In total, 48% fish regained feeding in HI treatment compared to only 31% fish in the LI treatment. More detailed analyses showed differences between the three groups of fish in each tank. In the tank exposed to HI, 63% and 69% fish had feed in intestine among AEFishBIT tagged fish and P-disc tagged fish, respectively. In the LI treatment, only 38% of individuals in both tagged groups had feed in their intestine, as opposed to 62% of animals with empty guts. Most of the control fish (80%) that were not tagged had an empty gut on inspection, while only 20% ate during the experimental period.

Atlantic salmon showed no signs of vertebral and jaw deformities, operculum shortage, snout damage, or emaciation. The most frequent damages for both tagged groups (P-disc and P-disc and AEFishBIT tagged fish) before and after the experiment were eye, skin, and fin damage (Table 2). The prevalence of eye damage among P-disc and AEFishBIT tagged fish was low (4%) at the beginning of the experiment but increased at the end (38%). The same trend was observed for fish tagged with P-disc only, where the percentage of fish with eye damage increased from 25% at the beginning of the experiment to 52% at the end of the trial. Most fish had skin damage at the beginning, and the prevalence increased up to 100% in both tagged groups. The prevalence of all fin damage also increased during the study for both tagged groups.

Analysis of individual severity of OWI scores over time showed that between 19% and 38% of individuals had an increase in severity of damage, apart from 65% of fish tagged with only P-disc, who showed an increase in skin damage (Table 3). The main reason for the increased severity of individual damage may be related to frequent handling of fish in a short period of time. On the basis of the OWI status of both tagged groups, we did not observe any additive effect of tagging with AEFishBIT compared to animals only tagged with P-disc.

## 4. Discussion

The present study indicates that the miniaturized AEFishBIT biosensor can be used successfully to monitor swimming activity and metabolic condition of Atlantic salmon in land-based aquaculture systems. This device was successfully used to monitor the performance of gilthead sea bream and European sea bass under different experimental conditions after monel piercing tagging [18,19,20,21]. However, the results of the present study revealed that this tagging procedure was not suitable for salmon. In fact, this tagging method caused visible damage to the gills (lamellae tissue loss and discoloration) and operculum (irritation) one week after tagging in the pilot study with dummy devices. Current studies with rainbow trout showed similar results (unpublished results), probably due to fish species differences in head shape and operculum ossification [24]. Therefore, it was necessary to test a new tagging method that took these distinctive features into account. The proposed solution was based on the use of suture and polyethylene tube that showed no visible negative effects on salmon. It is noteworthy that the retention rate of the biosensor in the pilot study was high (85%) after one week at a culture density of 27 kg·m^−3^, which is in the range of relevant densities for Atlantic salmon production in cages. Further verification will be needed for land-based related research, where Atlantic salmon densities can exceed 50 kg·m^−3^. However, there is potential to improve the tag retention rate by placing tags on the left operculum. This location would reduce the contact between the tag and the tank wall considering the counter clock swimming direction, which could have caused the tags to fall off. Otherwise, the use of a more appropriate fish size (greater than 350 g), a thicker suture, and a placement closer to the edge of the operculum could also be considered. For instance, the retention rate in the light regime experience was increased up to 98% at 4 days post-tagging with the use of larger fish (760 g in average). In the case of gilthead sea bream, the limitation of the size of the fish depending on the weight of the device is close to the 2% rule, which makes it possible to monitor fish of 100–300 g without pathological signs of gill hemorrhage, operculum damage, or changes in conventional blood stress markers 7–10 days post-tagging [18,20]. The general idea is that the 2% rule applies to internally or dorsally attached devices [25], but in the case of gilthead sea bream, the same threshold level is valid for operculum attached devices. In any case, large future improvements in the miniaturization of electronic devices are expected in coming years, which will allow easier smart sensing of farmed fish from earlier life stages.

Analysis of external morphological OWI during the light intensity challenge further corroborated that the proposed method for AEFishBIT tagging had no negative effects on post-smolt salmon, as there were no differences between the AEFishBIT and P-disc tagged groups. The increased prevalence of damage to eyes, skin, and fins was attributed to repeated handling of animals in a short period of time (twice in 4 days) with no observation of the most severe damages (score 3) [7]. Regardless of this, the existence of species-specific AEFishBIT tagging protocols highlights the importance of finding the best way of device attachment for each fish species. 

Tagging procedures for animal monitoring often involve some stress that affects the physiology and behavior of the fish until recovery [26,27]. Therefore, it is important to determine the minimum time elapsed until recovery of normal behavior to obtain reliable monitoring results. Atlantic salmon responds with increased swimming activity in stressful situations, such as irregular feeding or reduced tank flow [16]. In this study, the behavior of tagged post-smolt salmons showed a similar pattern with four times greater physical activity after immediate fish handling, with a relatively rapid reestablishment of the steady state 8 h post-tagging with the surgical thread protocol. This stabilization of physical activity is informative of the time it takes for the salmon to recover from handling and AEFishBIT tagging. A much longer time, between 4 and 6 days, was required after intraperitoneal implantation of electronic transmitter tags that measure activity and heart rate in adult salmon [28]. The differences observed are likely due to the more severe tagging procedures required for implantation of the intraperitoneal device. Considering the close link between heart rate and gill breathing [29], the use of external devices such as AEFishBIT is emerging as a real alternative to avoid the use of tedious and aggressive procedures for monitoring metabolic activity in free-swimming fish for short time periods.

In our experimental setup, the 2 day recording schedule was able to detect the transient increase in physical activity at the time the light regime was changed to HI conditions, a fact that was later confirmed by the video recording analysis. This correlation between AEFishBIT and video analysis results served as a functional validation of AEFishBIT measurements in these animals, confirming the potential of this device to monitor activity and overall performance in acute or short-term behavioral studies in Atlantic salmon. Thus far, monitoring of swimming activity is typically performed using camera technology or surgically implanted tags [7,10,11,16,28]. The use of AEFishBIT could help establish a quantitative behavioral analysis in an OWI [7]. In addition, it can improve the efficiency of behavioral studies by reducing the time spent on analyses and allowing monitoring for longer periods of time. It can also increase the application to production systems with turbid water (such as recirculation aquaculture systems) where use of camera technology is still limited.

The continuous light and continuous feeding conditions used in this study reflected the commercial rearing condition of Atlantic salmon. One of the consequences was the lack of rhythmicity in physical activity and respiratory frequency of tagged post-smolts, which was clearly established with the AEFishBIT recording. In any case, the noteworthy finding is that the mean respiratory frequency of salmon was in the range of 2.2–2.4 breaths·s^−1^. These values are consistently higher than those reported for gilthead sea bream or European sea bass (1.7–1.9) in free-swimming conditions [19]. Since the respiratory frequency can be considered an indirect indicator of basal metabolism [21], the higher value of salmon clearly reflects the large growth potential of this highly cultured species. This feature could be of utility in breeding programs and when considering prospective studies of new fish species for aquaculture.

Current studies in gilthead sea bream clearly indicate that changes in the respiration/activity ratio are highly informative of the different energy partitioning between growth and locomotor activity on a seasonal and daily basis (unpublished results). This also applies when making comparisons between gilthead sea bream and European sea bass, a well-known fast swimming predator [19]. The range of individual variation of this quotient remains poorly explored, but it is notable that gilthead sea bream strains selected for fast growth shared an increased contribution of aerobic metabolism to the whole energy demand for growth and locomotor activity [21]. Furthermore, in Atlantic salmon, the coefficient of variation for the calculated respiration/activity ratio can be used as a quantitative indicator of the adaptive responses to challenging conditions (e.g., lighting regime). Certainly, this metabolic index in C and HI groups was quite constant, while LI group showed larger variability, which may be indicative that fish subjected to lower light intensities have not yet fully adapted to these culture conditions after 4 days. This finding is reinforced when we consider that 62% of fish with AEFishBIT in LI group did not have any feed in their gut at the end of the experiment. In fact, the appetite of the post-smolt in this study was negatively affected, and video recording analysis confirmed that a large portion of individuals (regardless of tag presence/absence) in all treatments did not show interest in feed. Since we exposed salmon to repetitive stresses (handling, tagging, new spaces, and lighting changes), this response could be expected, as observed in earlier studies in which repeated stress reduced the appetite and growth of Atlantic salmon [30,31]. Further optimization of tagging protocols and prolonged use of tags with increased battery capacity, as well as future improvements in device miniaturization, will alleviate some of the main side effects of tagging and increase the usability of AEFishBIT under research and commercial conditions.

## 5. Conclusions

AEFishBIT can be used for the simultaneous and easy monitoring of physical activity and respiratory frequency from post-smolt life stage in Atlantic salmon. The results fitted well with analysis of video recording data, as well as the general patterns of swimming activity previously established with commercially available tags. The use of AEFishBIT becomes especially valuable for precise and rapid studies, pointing out individual differences in adaptation to handling/changing production conditions in post-smolt salmon.

## Figures and Tables

**Table 1:** Light intensities measured in the three tanks at three depths and average values.

Depth (m)	High Intensity (HI) (μmol·m^−2^·s^−1^)	Control (C) (μmol·m^−2^·s^−1^)	Low Intensity (LI) (μmol·m^−2^·s^−1^)
0	24.81	12.66	0.56
0.33	17.52	8.80	0.37
0.72 ^1^	14.40	6.83	0.28
Average light intensity	18.90	9.43	0.40

^1^ Tank bottom.

**Table 2:** Prevalence of damage of evaluated external morphological operational welfare indicators (OWI) recorded at the start of the light intensity experiment (before tagging) and at the end of experiment. In total, 48 individuals in both tagged groups (P-disc and AE-FishBIT with P-disc) were evaluated prior to tagging and at the end of experiment.

External Morphological OWI	Prevalence of Damage (% Individuals)			
	Start of Experiment		End of Experiment	
	P-Disc	AEFishBIT and P-Disc	P-Disc	AEFishBIT and P-Disc
Eye damage	25	4	52	38
Skin damage	77	98	100	100
Dorsal fin damage	50	75	54	85
Caudal fin damage	58	58	75	83
Pectoral finn damage	58	81	65	96
Pelvic fin damage	56	63	75	77

**Table 3:** Change in the severity of damage of evaluated operational welfare indicators prior to start and the end of the experiment. The numbers in the table represent percentages of fish with either worsened or improved/unchanged severity of damage (score between 0 and 3).

Tagged Group	Δ OWI over Time	Prevalence (% Individuals)					
		Eye	Skin	Dorsal Fin	Caudal Fin	Pectoral Fin	Pelvic Fin
P-disc	Deteriorated	35	65	23	35	25	35
	Improved/unchanged	65	35	77	65	75	65
AEFishBIT and P-disc	Deteriorated	38	19	27	35	25	31
	Improved/unchanged	62	81	73	65	75	69

## Data Availability

Data are available from authors on reasonable request.

## Supplementary Materials

The following are available online at https://www.mdpi.com/article/10.3390/ani11082403/s1, Figure S1. Positioning of the fish tagged with AEFishBIT in the tank with non-tagged Atlantic salmon. Figure S2. (A) Latency time (s) to when fish showed interest in feed pellets was monitored on D1. Data are shown as means ± SD. NS = not significant. NC = 24, NLI = 16, NHI = 24. (B) Proportion (%) of fish showing no interest in feed during observed period on D1.
