# Supplementary material for: A Novel Miniaturized Biosensor for Monitoring Atlantic Salmon Swimming Activity and Respiratory Frequency
Source: Animals (Basel). 2021 Aug 14;11(8):2403. doi: 10.3390/ani11082403 (PMC8388786; doi:10.3390/ani11082403)
Supplement: Supplementary file 1 [file animals-11-02403-s001.zip › animals-1301263-supplementary.pdf]

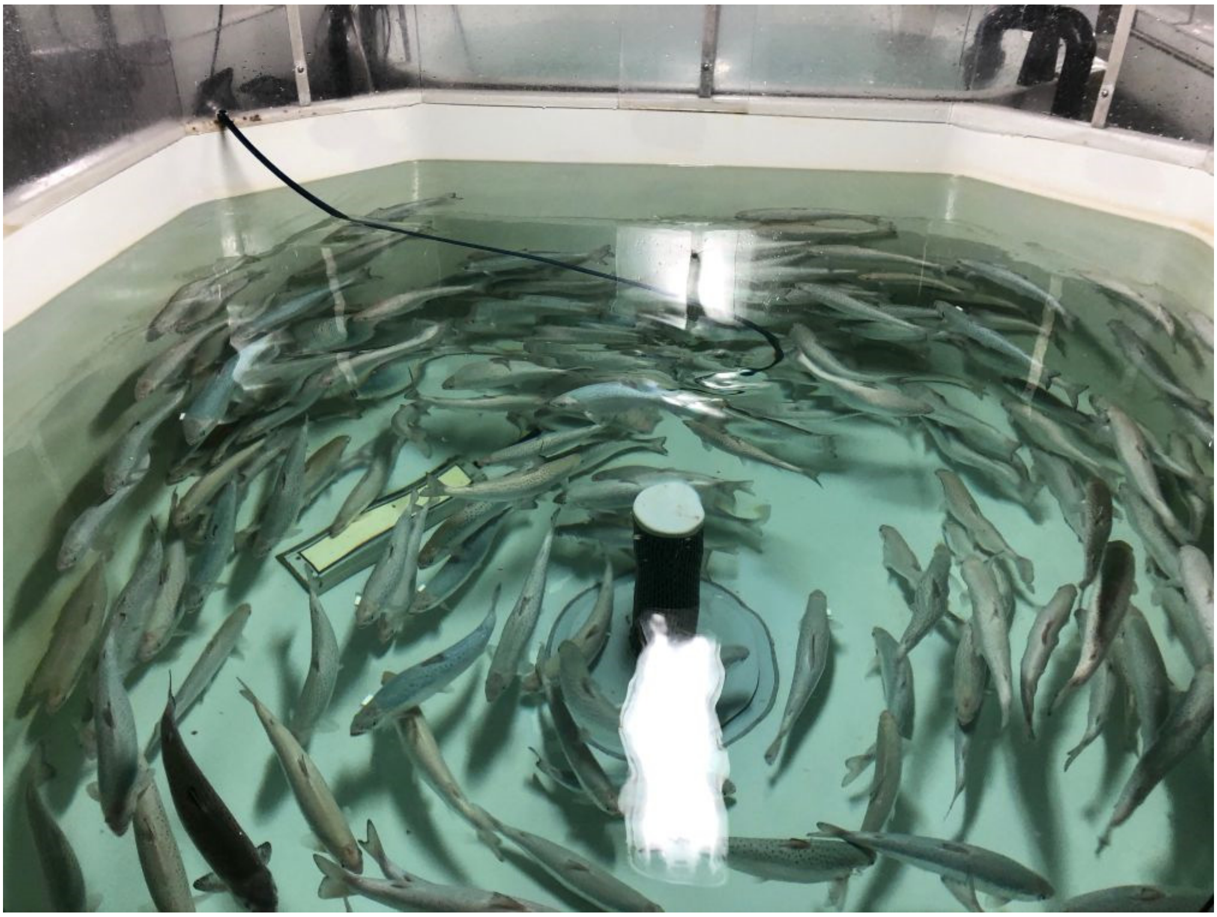

**Figure S1.** Positioning of the fish tagged with AEFishBit in the tank with non-tagged Atlantic salmon.

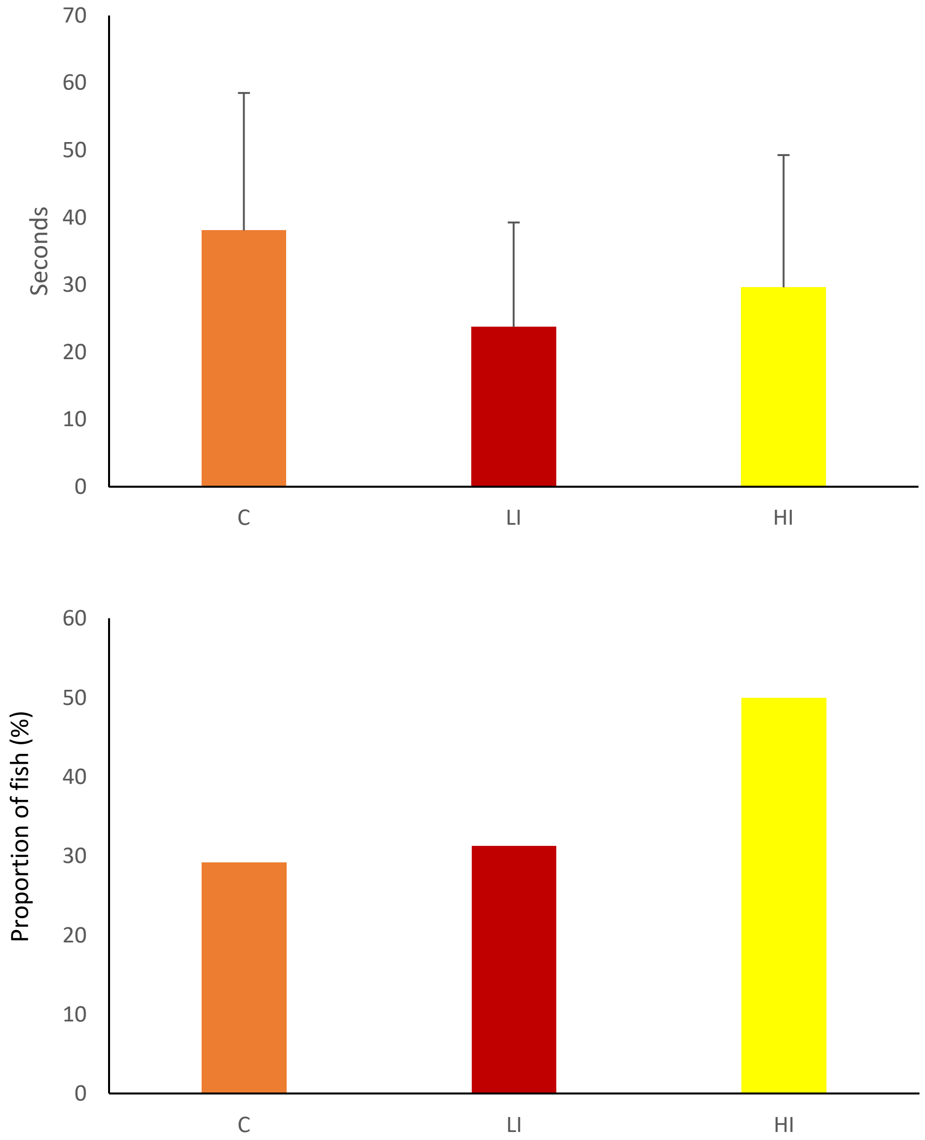


(A)

(B)

**Figure S2.** (**A**) Latency time (sec.) to when fish showed interest in feed pellets was monitored on D1. Data is shown as means ± SD. NS = Not significant. $N_{C}$ = 24, $N_{LI}$ = 16; $N_{HI}$ = 24. (**B**) Proportion (%) of fish showing no interest in feed during observed period on D1.
